# Radical‐Mediated Degradation of Thiol–Maleimide Hydrogels

**DOI:** 10.1002/advs.202402191

**Published:** 2024-04-06

**Authors:** Tayler S. Hebner, Bruce E. Kirkpatrick, Benjamin D. Fairbanks, Christopher N. Bowman, Kristi S. Anseth, Danielle S.W. Benoit

**Affiliations:** ^1^ Department of Bioengineering University of Oregon 6231 University of Oregon Eugene OR 97403 USA; ^2^ Department of Chemical and Biological Engineering University of Colorado Boulder 596 UCB Boulder CO 80309 USA; ^3^ BioFrontiers Institute University of Colorado Boulder 596 UCB Boulder CO 80309 USA; ^4^ BioFrontiers Institute Medical Scientist Training Program University of Colorado Anschutz Medical Campus 13001 East 17th Place Aurora CO 80045 USA

**Keywords:** degradation, hydrogels, Michael addition, photopatterning, radicals

## Abstract

Michael addition between thiol‐ and maleimide‐functionalized molecules is a long‐standing approach used for bioconjugation, hydrogel crosslinking, and the functionalization of other advanced materials. While the simplicity of this chemistry enables facile synthesis of hydrogels, network degradation is also desirable in many instances. Here, the susceptibility of thiol–maleimide bonds to radical‐mediated degradation is reported. Irreversible degradation in crosslinked materials is demonstrated using photoinitiated and chemically initiated radicals in hydrogels and linear polymers. The extent of degradation is shown to be dependent on initiator concentration. Using a model linear polymer system, the radical‐mediated mechanism of degradation is elucidated, in which the thiosuccinimide crosslink is converted to a succinimide and a new thioether formed with an initiator fragment. Using laser stereolithography, high‐fidelity spatiotemporal control over degradation in crosslinked gels is demonstrated. Ultimately, this work establishes a platform for controllable, radical‐mediated degradation in thiol–maleimide hydrogels, further expanding their versatility as functional materials.

## Graphical abstract

Thiol–maleimide Michael addition reactions are often employed in the synthesis of hydrogels. In this work, radical‐mediated degradation of the Michael adducts formed between thiol and maleimide‐functionalized poly(ethylene glycol) macromers is reported. Using both redox‐ and photo‐initiated radicals, this common hydrogel formulation is shown to have rapid and spatiotemporally tunable degradability.

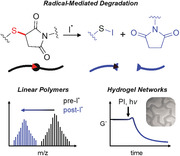

## 1 Introduction

Synthetic hydrogels are ubiquitous in many applications, from tissue engineering scaffolds to cosmetics and textiles, agricultural soil treatments, and wastewater purification.^[^ ^1^ ^]^ Their versatility is largely attributed to the immense design space that enables material property control for a wide range of application‐driven functionalities, starting from non‐toxic components. Targeted functional properties are realized by selecting underlying monomer and macromer components with reactive end‐groups that enable the formation of a crosslinked network. In many instances, end groups that facilitate click‐like reactions are leveraged for maximal speed, conversion, and selectivity. Some examples used extensively in hydrogel chemistry include radical‐mediated thiol‐ene, thiol–Michael addition, and strain‐promoted azide–alkyne reactions.^[^ ^2^, ^3^ ^]^ In particular, the Michael‐type addition reaction of a thiolate end‐group with an electron‐deficient alkene has become a dominant chemistry in hydrogel formation due to mild reaction conditions necessary for applications such as tissue engineering, where crosslinking must often be carried out at physiological pH and temperature.^[^ ^4^, ^5^, ^6^ ^]^ Unlike Michael‐type reactions with alkenes such as acrylates and vinyl sulfones, the thiol–maleimide chemistry does not typically require the addition of a base catalyst or elevated temperature for complete conversion of the functional groups.^[^ ^5^ ^]^ As such, the simplicity of the thiol–maleimide chemistry provides an especially facile approach to the design of materials with well‐defined structures and properties.

Thiol–maleimide hydrogels are favorable for many applications owing to their generally stable network structure, allowing long‐term studies.^[^ ^7^ ^]^ However, degradability in hydrogel networks is sometimes desirable and, in certain cases, critical to their application. The flexibility of macromer structure in synthetic thiol–maleimide hydrogels facilitates the incorporation of multiple forms of degradable bonds.^[^ ^8^ ^]^ Notably, these hydrogels must be designed with degradation mechanisms that are sensitive to stimuli orthogonal to those necessary for network formation or other responsive properties. One class of degradable materials that has been commonly employed is networks with irreversibly cleavable bonds. In tissue engineering applications, enzymatically degradable bonds have been leveraged to enable the remodeling of the hydrogel in a manner similar to what is observed in the native extracellular matrix.^[^ ^9^, ^10^, ^11^, ^12^ ^]^ This approach commonly employs matrix metalloproteinase (MMP)‐cleavable peptide sequence‐based linkers flanked with thiols, which are often cysteine residues. Another class of degradable bonds that have been incorporated into these hydrogels is hydrolytically cleavable bonds, such as esters. Depending on the type of Michael acceptor used for crosslinking, hydrolysis in thiol–Michael hydrogels is often inherent to the crosslink structure, enabling tunability of network properties to span from fast‐degrading to highly stable materials. For example, thioethers formed with acrylates readily undergo network‐degrading ester hydrolysis, while maleimide‐derived thiosuccinimides are generally more stable.^[^ ^13^ ^]^ Integrating irreversible degradation modes into hydrogels has been instrumental in advancing several research areas, as seen prominently in tissue engineering with the advent of synthetic scaffolds, which can gradually be replaced by matrix deposition and proliferation of their encapsulated cells or host cells.^[^ ^14^ ^]^

Additional considerations in designing degradable hydrogels are the rate and localization of degradation in the material. For example, in MMP‐degradable materials, the degradation rate is controlled by kinetics and diffusion parameters related to the concentration and type of MMP secreted by hydrogel‐encapsulated and host cells. The degradation rate is easily controlled via pH or temperature in supramolecular systems, but spatial localization is challenging. In hydrolytically degradable hydrogels, rate is controlled by the substituents on the cleavable bonds and the solvent conditions of the hydrogel including pH and temperature. Still, localization is also challenging in these systems.^[^ ^15^, ^16^, ^17^, ^18^, ^19^ ^]^ To enable true spatiotemporal control of degradation, numerous approaches have been taken to integrate degradability facilitated by light as a stimulus.^[^ ^20^, ^21^, ^22^, ^23^, ^24^ ^]^ Light is particularly amenable for controlling degradation rate via intensity or wavelength (i.e., absorbance) and can be localized by structured illumination with sub‐micron precision. As such, many degradation mechanisms utilize photoresponsive or photocleavable moieties to enable advanced applications, such as spatially directed cell growth or drug delivery.^[^ ^25^, ^26^ ^]^

In the approaches discussed thus far, degradability is introduced in thiol–maleimide hydrogels by incorporating additional functional groups in the monomer or macromer structure. However, dissociation of the Michael adducts themselves is a less explored, but versatile strategy for the design of degradable hydrogels. Retro‐Michael reactions can reverse Michael adducts to their constituent thiol and alkene moieties, but the aqueous microenvironment of hydrogels typically favors Michael adduct stability.^[^ ^7^, ^27^ ^]^ Therefore, alternative mechanisms are necessary to achieve facile degradation of these materials. Prior demonstrations using both small molecules and polymers have shown the potential for radical‐mediated bond cleavage of C─S bonds.^[^ ^28^ ^]^ Carbon‐centered radicals are generated from these C─S bonds via homolytic substitution, with the form of radical generation strongly dependent on the substituents of the carbon and sulfur atoms. In recent work, this mechanism of radical substitution has been leveraged in thiol‐ene polymers containing C─S bonds to enable dynamic network behavior in the form of reversible gelation.^[^ ^29^, ^30^ ^]^ While these thiol‐ene polymers demonstrated the generation of primary C‐centered radicals, secondary carbon radicals have comparatively higher stability.^[^ ^31^ ^]^ Therefore, secondary C‐centered radical formation from C─S bonds involving tertiary carbons, such as the Michael adducts formed in thiol–maleimide hydrogels, is likely to occur in a radical‐rich environment. Additionally, since radical generation is amenable to numerous stimuli, including light, the radical‐mediated cleavage of C─S bonds could enable spatiotemporal control of degradation in these materials and prove favorable in the design of orthogonal reactions. However, no studies to date have explored the potential for radical‐mediated degradation of thiol–Michael hydrogels in the absence of other radical‐cleavable functional groups.^[^ ^32^ ^]^

In this work, we demonstrate the formation of conventional thiol–maleimide hydrogels in the presence of a radical photoinitiator followed by degradation of the crosslinked networks upon radical generation. Initial studies were conducted using photo‐initiated radicals to simplify the analysis of degradation by leveraging the temporal control of light as an applied stimulus, and we extend these findings to a depth‐independent redox radical initiator system. Linear starting materials were used to form polymers that also exhibited radical‐mediated degradation. These linear systems were used to identify degradation products, finding that radical‐mediated degradation occurs at the thioether bond between the sulfur and beta carbon of the Michael adduct. Leveraging the spatial control of light, this degradation mechanism was used to demonstrate selective patterning of poly(ethylene glycol) (PEG)‐based thiol–Michael hydrogels via primary radicals from photoinitiator. Taken together, these results illustrate the potential for radical‐mediated degradation of thiol–maleimide polymers to be extended in a broad range of future applications. The discovery of this degradation mechanism highlights the importance of hydrogel crosslink design when incorporating multiple stimuli (radicals, pH, etc.) for orthogonal modulation of functionalities such as cell behavior, hydrogel mechanics, or stimuli‐responsive properties.

## 2 Results and Discussion

**Figure 1:**
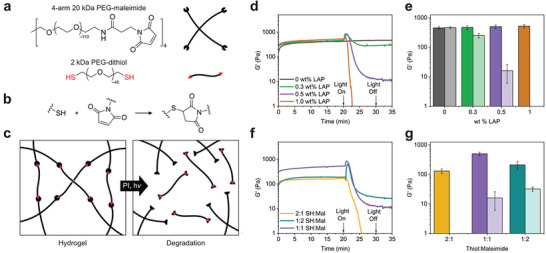
Thiol–maleimide hydrogel synthesis and photodegradation. a) Poly(ethylene glycol)‐based multifunctional thiol and maleimide macromers were used to synthesize crosslinked networks in PBS containing 5 wt.% macromer and LAP as a photoinitiator (0.5 wt.% unless noted). b) Network formation occurs via thiol–Michael reaction between thiol and maleimide end‐groups, forming a thiosuccinimide adduct. c) Crosslinked networks are irreversibly degraded by photoinitiated radical generation. d) Network formation and degradation were monitored by in situ oscillatory shear rheology (1% strain, 1 rad/s) for hydrogels with varied LAP concentration and f) varied thiol:maleimide molar ratios. Michael addition was allowed to proceed for 20 min, followed by 10 min of 365 nm light exposure (5 mW cm^−2^) and 5 min in the dark. Storage moduli of the hydrogel after Michael addition (dark bars) and after UV exposure (light bars) are compared for e) varied LAP concentration and g) varied thiol:maleimide molar ratios.

**Figure 2:**
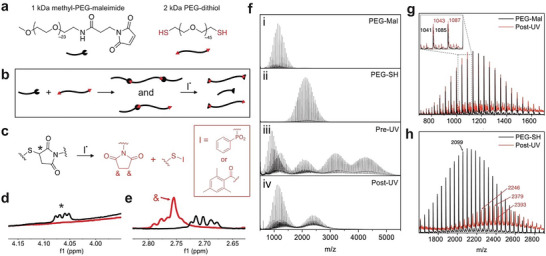
Characterization of degradation products using linear polymers. a) 2 kDa PEG‐dithiol and 1 kDa mPEG‐maleimide are used as starting materials. b) Michael addition of starting materials forms linear polymers that are subsequently degraded as a result of photoinitiated radical generation, resulting in c) the Michael adduct degrading to the products shown in red. ^1^H NMR spectra of Michael‐addition adducts (black) and degradation products (red) show d) the presence of the thiosuccinimide (*) before UV exposure and e) the presence of succinimide (&) after degradation. f) MALDI spectra of the mPEG‐maleimide i) and PEG‐SH ii) starting materials, the Michael‐addition products iii), and degradation products iv). MALDI spectra of degradation products (red) are compared to the starting materials (black) for g) 1 kDa and h) 2 kDa ranges, supporting the mechanism shown in panel (c).

A thiol–maleimide hydrogel was synthesized via thiol–Michael addition using a linear 2 kDa poly(ethylene glycol) (PEG) dithiol macromer in combination with a four‐arm maleimide‐functionalized PEG macromer (**Figure** **1**a). Macromers were mixed at a thiol:maleimide molar ratio of 1:1 in phosphate‐buffered saline (PBS) at 5 wt.% macromer (9 mm functional groups) in combination with 0.5 wt.% (17 mm) photoinitiator (lithium phenyl‐2,4,6‐trimethylbenzoylphosphinate, LAP). The Michael addition reaction (Figure 1b) was allowed to proceed for 20 min to form the final crosslinked network (Figure 1c). This reaction was monitored in situ via oscillatory shear rheology (1 rad/s, 1% strain) over 20 min at room temperature to monitor the progress of the polymerization reaction prior to photoinitiated network degradation (Figure 1d). To study radical‐mediated degradation, the hydrogels were exposed to UV light (365 nm, 5 mW cm^−2^) for 10 min, followed by 5 min. in the dark. In the presence of a photoinitiator, a transient increase in the storage modulus (G’) was observed, followed by a substantial decrease, attributable to a reduction in crosslink density via radical‐mediated degradation. After 10 min. of UV exposure, G’ was markedly lower in magnitude than that of the gel before exposure, except for the 0 wt.% LAP condition (Figure 1e). Hydrogels maintained their final moduli once light was removed, indicating an irreversible degradation mechanism. The pre‐and post‐UV moduli are compared in **Table** **1**. Irreversible radical‐mediated degradation was further validated by exposing a hydrogel containing LAP to UV light at regular intervals, resulting in stepwise degradation (Figure S1, Supporting Information). No degradation or formation of new bonds occurred during the dark intervals when no new radicals were being generated, as indicated by the constant storage modulus. To determine if the observed behavior was applicable to Michael adducts with alternative thiol substituents that alter their reactivity, a cysteine‐functionalized peptide crosslinker was substituted for the PEG‐dithiol, and similar radical‐mediated degradation was observed (Figure S2, Supporting Information). Notably, prior studies have confirmed that non‐thiol amino acid residues do not participate in radical‐mediated hydrogel synthesis (i.e., thiol‐ene reactions), suggesting that the degradation mechanism proposed here would be isolated to the thiol–maleimide adduct similar to non‐peptide containing gels.^[^ ^33^ ^]^ Given the observed changes in modulus and evident degradation of the thiol–maleimide hydrogels, we next sought to investigate mechanistic aspects of the degradation process. The impact of initiator concentration, and therefore the number of generated radicals, on degradation behavior was investigated to further explore radical‐mediated degradation in these hydrogels. In the materials synthesized here using 4‐functional and 2‐functional macromers to form a network at equivalent stoichiometry, Flory‐Stockmayer theory predicts that ≈42% of crosslinks must be cleaved for reverse gelation. Based on a conservative estimated quantum yield of 0.5 for LAP,^[^ ^34^ ^]^ sufficient radical generation to cleave >50% of Michael adducts at 5 mW cm^−2^ would occur within 100 s in an optically thin material, which falls within the timespan of degradation in the experimental data. Full theoretical calculations and assumptions are found in the Supporting Information. To investigate degradation relative to the efficiency of radicals in degrading crosslinks (versus side reactions) in experimental conditions, hydrogels were synthesized with a 1:1 molar ratio of thiol:maleimide groups and varying concentrations of photoinitiator and performed in situ shear rheology experiments to monitor changes in the crosslinking density during degradation (**Figure** **2**b). For a LAP concentration of 0.3 wt.%, the number of radicals generated was approximately equivalent to the number of Michael adducts (≈10 mm radicals vs 9 mm crosslinks), leading to the expectation that the optically thin material would fully degrade. However, incomplete degradation was observed after UV exposure. This incomplete degradation was also observed with an initiator concentration of 0.5 wt.%, where the number of expected radicals exceeds the number of Michael adducts (17 mm radicals). This material exhibited a more substantial decrease in the final modulus, suggesting that more degradation events occurred by increasing the number of generated radicals. Finally, the hydrogel fully degraded when the initiator concentration was increased to 1 wt.% (34 mm radicals, greater than a two‐fold excess of radicals relative to Michael adducts). Loss moduli are plotted alongside storage moduli to show the extent of degradation (Figure S3, Supporting Information).

**Table 1:** Storage modulus of hydrogels after the Michael addition reaction (20 minutes), peak storage modulus after initial UV exposure (365 nm, 5 mW/cm^2^), and the final storage modulus of the hydrogel after 10 minutes of UV exposure with four‐arm PEG‐maleimide and 2 kDa PEG‐dithiol (5 wt% total macromer) using varied photoinitiator (LAP) concentrations and thiol:maleimide ratios.

wt.% LAP	SH:Mal	Radicals: Crosslinks	Pre‐UV Modulus^a)^ [Pa]	Peak Modulus^a)^ [Pa]	Post‐UV Modulus^a^ ^)^ [Pa]
0	1:1	0	456 ± 50	‐	460 ± 40
0.3	1:1	1.1	470 ± 70	800 ± 100	250 ± 40
0.5	1:1	1.9	500 ± 70	780 ± 70	16 ± 10
1.0	1:1	3.8	521 ± 40	700 ± 120	‐
0.5	2:1	1.9	180 ± 20	180 ± 20	‐
0.5	1:2	1.9	210 ± 60	540 ± 160	32 ± 5

^a)^ Measured using oscillatory shear at 1 rad/s, 1% strain.

Considering the initial increase in storage modulus upon UV exposure, we hypothesized that the discrepancy between the statistical‐kinetic model of the reverse gel point and those measured experimentally could be attributed to kinetically favored maleimide crosslinking due to incomplete conversion in the initial thiol–Michael addition reaction.^[^ ^35^ ^]^ To test this possibility, off‐stoichiometry gels were synthesized using molar ratios of thiol:maleimide of 1:2 and 2:1, and the resulting materials were again subjected to in situ oscillatory shear rheology (Figure 1f). Excess thiol resulted in no initial increase in storage modulus upon initial UV exposure and complete degradation after 5 min of exposure, as indicated by the crossover of loss modulus (Figure S4, Supporting Information). Comparatively, excess maleimide resulted in a larger initial increase in modulus with UV exposure than networks formed with equimolar functional groups, and the final modulus of the material plateaued at a value closer to that of the pre‐UV material. We interpret these results to support that radical‐mediated crosslinking between maleimides drives a transient, short‐term increase in network crosslinking (G’) until radical‐mediated degradation dominates over longer timescales. Therefore, while the calculations in the prior discussion assume that all radicals participate in degradation events, kinetically favored maleimide crosslinking events also consume radicals in these hydrogel formulations. Collectively, these results suggest that initiator concentration, light exposure time, and the initial stoichiometric ratio of thiol:maleimide could all be tailored to accommodate both radical‐mediated crosslinking/stiffening (i.e., short exposure time) or degradation/softening (i.e., longer exposure times) all in proportion to the amount of pendant, unreacted maleimides. Taken together, these experiments demonstrate a tunable radical‐mediated degradation process, spanning from controlled stiffening and softening to full degradation of thiol–maleimide hydrogels. While in situ shear rheology indicated radical‐mediated degradation of thiol–maleimide networks, studying only crosslinked networks limited our ability to determine the underlying degradation mechanism. As such, a model linear polymer system was synthesized from monofunctional mPEG‐maleimide and bifunctional PEG‐thiol (**Figure** **2**a). The macromers were dissolved in PBS at a 1:1 molar ratio of thiol:maleimide at functional group concentrations equivalent to the crosslinked gels (9 mm) with 0.5 wt.% LAP. As before, the Michael addition reaction was allowed to proceed for 20 min to form linear polymers of either dimers or trimers before being exposed to UV light (365 nm, 5 mW cm^−2^, 15 min) to degrade Michael adducts (Figure 2b,c). NMR spectra were collected before and after photoinitated radical generation (Figure 2d,e). A Michael adduct between the thiol and maleimide macromers was observed as the proton on the tertiary carbon of the thiosuccinimide adduct, which disappeared following UV exposure (Figure 2d). Furthermore, a singlet near 2.76 ppm appeared after UV exposure, likely corresponding to a succinimide product (Figure 2e).^[^ ^36^ ^]^ Full NMR spectra are shown in the Supporting Information (Figure S5, Supporting Information). It should be noted that when conducting these experiments, a white precipitate formed after UV exposure in PBS. Since the observed amount of precipitate increased with photoinitiator concentration, the precipitate was hypothesized to be associated with initiator fragments. To avoid the inadvertent experimental impact of insoluble degradation products, a secondary system using trimethylbenzoyl diphenyl phosphine oxide (TPO) as the photoinitiator was studied in CDCl_3_, in which all products remained soluble. The NMR peaks of interest in both pre‐UV and post‐UV samples were similar to those seen in the LAP/PBS systems but with additional peaks that are attributed to the initiator fragments (Figure S6, Supporting Information). Therefore, these results support the proposed mechanism of radical‐mediated degradation of the thiol–maleimide adduct.

To characterize the degradation products further, the molecular weight of the linear polymers was analyzed using MALDI, before and after UV exposure in the presence of LAP. The products of the Michael addition reaction between 2 kDa mPEG‐maleimide (Figure 2f‐i) and 1 kDa PEG‐SH (Figure 2f‐ii) consisted of dimers comprised of one thiol macromer with one maleimide macromer and trimers of two maleimide macromers with one dithiol macromer, as indicated by the center of the molecular weight distributions ≈3200 and 4200 kDa, respectively, in the MALDI spectrum (Pre‐UV, Figure 2f‐iii). Unreacted 1 kDa mPEG‐maleimide and 2 kDa PEG‐SH also appear in these spectra due to incomplete conversion in the Michael addition reaction, analogous to what was observed in the crosslinked polymer networks. After exposure to UV light (10 min, 365 nm, 5 mW cm^−2^), the molecular weight distributions shift to a bimodal distribution representing two fragments of the degraded polymer (Post‐UV, Figure 2f,iv). One portion of the bimodal distribution aligns with the 1 kDa mPEG‐maleimide starting material but shifted by m/z of 2, indicating the addition of two hydrogen atoms to the maleimide and the formation of a succinimide (Figure 2g). The second portion of the bimodal distribution aligns with the expected molecular weight for the 2 kDa PEG‐SH starting material combined with either one or two LAP initiator fragments (Figure 2h). The radical‐mediated degradation mechanism was further supported by exposing a sample with no LAP to UV light and analyzing the products, which showed no change in the starting polymer molecular weight or NMR spectra (Figure S7, Supporting Information).

Collectively, the analytical studies performed on the linear polymer system enabled the identification of the degradation products and the deduction of the likely mechanism. In this mechanism, an initiator fragment facilitates radical bond cleavage at the tertiary carbon of the thiosuccinimide, with the initiator fragment ultimately conjugated to the sulfur atom of the Michael adduct. A secondary carbon radical is formed on the maleimide side of the Michael adduct, which likely abstracts hydrogen from the solvent or another source (e.g., initiator fragment, ethylene glycol repeat unit, etc.) to produce the ultimate protonated succinimide product. This proposed mechanism is supported by prior literature detailing radical‐mediated substitution reactions of thioethers.^[^ ^28^ ^]^ When an initiating species attacks the tertiary carbon, the products are determined by relative radical stability; a carbon radical is more stable than a primary thiyl radical, and therefore the proposed mechanism agrees with this reported behavior.

**Figure 3:**
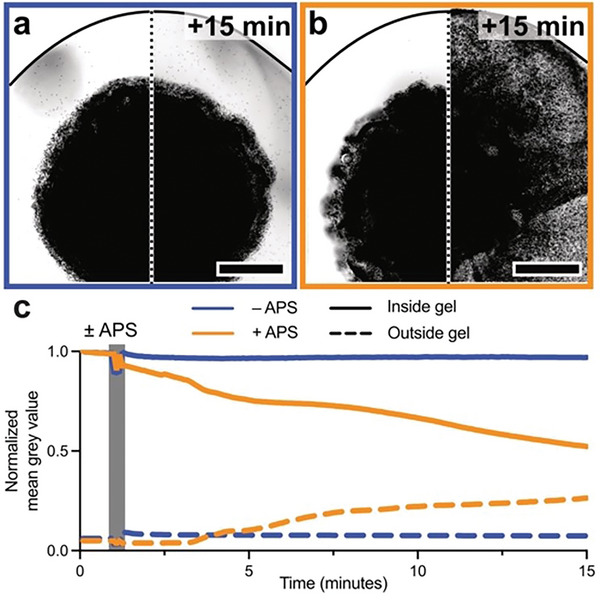
Radical‐mediated degradation using a chemical initiator system. 8 µL hydrogels (5 wt.% total polymer content) laden with 1 µm diameter fluorescent polystyrene beads were polymerized in a 96‐well plate, equilibrated in 0.2 m TEMED solution, and treated with an equal volume of either a) 0 m APS (PBS only, final concentration 0.1 m TEMED/0 M APS) or b) 0.2 m APS solution (final concentration 0.1 m TEMED/0.1 m APS). After 15 min, greyscale‐converted fluorescence imaging indicates no change in hydrogel bead localization in the –APS gel while the +APS gel degraded, as indicated by signal dispersion, suggesting bead release. Scale bars = 1 mm. The black line indicates a well boundary. c) Normalized mean grey values inside (solid lines) versus in solution outside (dashed lines) of bead‐laden hydrogels indicate rapid cargo release upon the addition of APS solution (orange) but not PBS only (blue).

**Figure 4:**
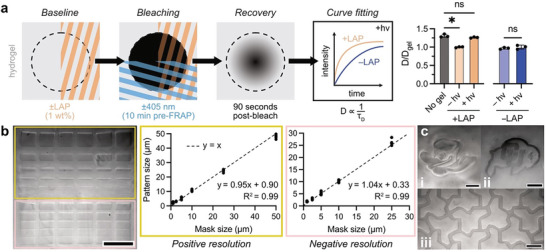
Spatially patterned degradation of thiol–maleimide hydrogels by photoinitiated radicals. a) Hydrogels were prepared for FRAP measurement with and without LAP (1 wt.%) and with and without 405 nm light patterning (10 min before fluorophore bleaching). Monitoring fluorescence recovery for 90 s and fitting the resulting intensity measurements yielded diffusion coefficients for each condition. Normalizing to fluorophore diffusion inside hydrogels before photodegradation, samples treated with LAP and 405 nm light had comparable diffusion to free fluorophore (a ≈25% increase in relative diffusion rate), suggesting total or near‐complete gel degradation. Samples treated with 405 nm light only (no LAP) exhibited no changes in diffusion. b) Resolution testing of photodegradation in a thiol–maleimide hydrogel showed high pattern fidelity for both positive (yellow boxes) and negative (pink boxes) features. Scale bar = 100 µm. c) Arbitrary subtractive photopatterning in thiol–maleimide hydrogels of the i) University of Oregon Duck and ii) University of Colorado Buffalo, as well as iii) an aperiodic monotile metamaterial, showing complex pattern retention following photodegradation. Scale bars = 50 µm.

Having elucidated the mechanism and products of degradation in thiol–maleimide hydrogels with photoinitiated radical generation, we next investigated if the degradation could be initiated more generally, by other radical‐generating initiators. To do so, 5 wt.% hydrogels containing fluorescent polystyrene beads (1 µm diameter) were prepared as 8 µL droplets in a 96‐well plate. These gels were equilibrated in a solution of 0.2 m tetramethylethylenediamine (TEMED) before the addition of an equal volume of either 0.2 m ammonium persulfate (APS) solution to initiate radical generation or PBS as a non‐radical generating control. For hydrogels treated with TEMED and PBS only, no network degradation or bead release was observed within 15 min (**Figure** **3**a). In contrast, hydrogels treated with APS/TEMED rapidly degraded and released the encapsulated beads over the same time period (Figure 3b), demonstrating the stability of the thiosuccinimide adduct in the presence of base but not free radicals. Monitoring bead release by time‐lapse fluorescence microscopy (Figure 3c), the onset of hydrogel degradation began immediately after the addition of APS, validating that redox initiator generated radicals also induce thiol–maleimide hydrogel degradation, even in optically thick samples or in materials containing photosensitive cargo. To examine the ability to spatially direct degradation with controlled illumination, thiol–maleimide hydrogels were exposed to 405 nm laser light and the extent and fidelity of degradation‐induced pattern transfer were characterized. First, fluorescence recovery after photobleaching (FRAP) was used to quantify the diffusion of a small molecule fluorophore (calcein, MW = 623 Da) before and after 405 nm patterning (**Figure** **4**a). In this experiment, fluorophore is bleached in a designated region, and subsequent radial diffusion of unbleached fluorophore from surrounding areas results in a recovery of fluorescence intensity measured in the bleached region. Therefore, a degraded hydrogel would be expected to display faster recovery of fluorescence based on lower crosslink density and improved diffusion. FRAP measurements were performed for free fluorophore (no gel) and 5 wt.% thiol–maleimide hydrogels containing either no or 1 wt.% LAP, with and without 405 nm patterning. Curve fitting of fluorescence recovery data in MATLAB gave characteristic diffusion times and diffusion coefficients. Normalizing calculated diffusion coefficients to measurements in non‐degraded hydrogels revealed recovery of free diffusion only in hydrogels containing LAP and irradiated with 405 nm laser light, consistent with the other results indicating degradation in the presence of radicals. Next, we tested the resolution limits of photopatterned degradation in 250 µm‐thick, 5 wt.% hydrogels containing 1 wt.% LAP and 2 µM FITC‐PEG 2 kDa‐thiol for gel visualization. A 405 nm laser was used to spatially pattern regions of degradation using a digital photomask with replicated positive and negative micron‐scale features (Figure 4b). High pattern fidelity was observed in both positive and negative features, with near‐perfect linear scaling (*R* ^2^ = 0.99) between digital mask and physical pattern dimensions for a range of 1–50 µm feature sizes. The actual pattern sizes were an average of 0.5–1 µm larger than the dimensions prescribed by the digital mask, suggesting diffusion of radicals. At smaller feature sizes, this diffusion of radicals results in a higher magnitude percent error in pattern fidelity (i.e., 50–100% error for 1 µm feature size, but only 1–2% error for 50 µm feature size). The ability of the thiol–maleimide formulation to retain complex patterns even with large fractions of degraded volume is further illustrated with the patterning of our university mascots (Figure 4c‐i,ii) and a metamaterial‐like aperiodic monotile^[^ ^37^ ^]^ (Figure 4c‐iii). These patterns were inscribed in the hydrogel matrix with high fidelity, demonstrating facile and spatiotemporal softening and degradation of this ubiquitous crosslinking chemistry.

Collectively, this work has established a new platform for stimulus‐controlled degradation of thiol–maleimide hydrogels. The radical‐mediated process results in irreversible cleavage of the C─S bond inherent to the product of Michael addition reactions, providing a facile degradation mechanism that maintains the simplicity of the thiol–maleimide hydrogel system and further expands its versatility. While this degradation mechanism was studied exclusively in the context of thiol–maleimide hydrogels and linear polymers, and we do not investigate this phenomenon in the presence of biological thiols or chain transfer agents, these findings have implications that extend to numerous other areas such as bioconjugations and surface functionalization for grafting via controlled radical polymerization.

## 3 Conclusion

These studies have elucidated a general mechanism for irreversible radical‐mediated degradation of thiol–maleimide polymers synthesized via Michael addition. Using crosslinked hydrogels, degradation via photoinitiated and chemically initiated radicals was demonstrated, with the extent of degradation depending on radical concentration. Degradation products were identified using linear polymers, finding that the radical‐mediated degradation of these materials produces the succinimide analog of the maleimide starting material and an initiator fragment covalently bound to the thiolated starting material. After confirming the general degradation mechanism in these materials, we used laser stereolithography to perform high‐fidelity patterning of degradation in thiol–maleimide hydrogels facilitated by photoinitiated radicals. In sum, this study introduces a facile method for spatiotemporal degradation in thiol–maleimide hydrogels, further expanding upon the versatility of this ubiquitous chemistry for advanced applications of hydrogel materials.

## 4 Experimental Section

### Synthesis and Degradation of thiol–Maleimide Hydrogels

Generally, hydrogels were synthesized using a 4‐arm 20 kDa poly(ethylene glycol) macromer functionalized with maleimide end‐groups (PEG‐Mal, Jenkem), Macromers were combined in a solution of 5 wt.% solids (9 mm crosslinks) in phosphate‐buffered saline (PBS) at thiol:maleimide molar ratios of 1:2, 1:1, or 2:1. Lithium phenyl‐2,4,6‐trimethylbenzoylphosphinate (LAP, synthesized or purchased from Sigma Aldrich) was incorporated as a photoinitiator at a concentration of 0, 0.3, 0.5, or 1 wt.%.

Hydrogels containing dicysteine functionalized peptide (KCGPQGIAGQCK, American Peptide) were synthesized by combining the peptide with the 4‐arm 20 kDa PEG‐Mal macromer at thiol:maleimide ratio of 1:1 with 0.5 wt.% LAP in PBS at a concentration of 5 wt.% solids.

For all formulations, after mixing the macromers and initiator in solution, the Michael addition reaction was allowed to proceed for 20 min at room temperature. For photodegradation, the gels containing LAP or no initiator were then exposed to UV light (365 nm, 5 mW cm^−2^) for 10 minutes.

### Synthesis and Degradation of Linear Thiol–Maleimide Polymers

Linear polymers were synthesized using 2 kDa PEG dithiol and monofunctional 1 kDa PEG‐maleimide at a thiol:maleimide molar ratio of 1:1. Macromers were combined in solution (PBS) at a 9 mm functional group concentration with 0.5 wt.% LAP or with no initiator. In one experiment, the macromers were combined in solution (CDCl_3_) at 9 mm functional group concentration with 0.5 wt.% trimethylbenzoyl diphenyl phosphine oxide (TPO, Sigma Aldrich). After combining in solution and vortex mixing, the Michael addition reaction was allowed to proceed for 20 min at room temperature. For photodegradation, the solution was then exposed to UV light (365 nm, 5 mW cm^−2^). Samples were flash‐frozen in liquid nitrogen and lyophilized overnight. Dried solids were then used to prepare appropriate solutions for the characterization experiments described below. The sample prepared with TPO in CDCl_3_ was not lyophilized, and instead was used directly in NMR experiments.

### Matrix‐Assisted Laser Desorption/Ionization Mass Spectrometry

Matrix‐assisted laser desorption/ionization (MALDI) mass spectrometry experiments were performed in linear mode at 95% laser power with a 337 nm, 60 Hz Nitrogen laser (Microflex Smart LS, Bruker) using a dithranol matrix (5 mg mL^−1^) and a sodium trifluoroacetate electrolyte (NaTFA, 1 mg mL^−1^). NaTFA solution (1 mg mL^−1^) was mixed in equal parts with the polymer solutions (5 mg mL^−1^). Acetonitrile was used as a solvent for the dithranol, and water was used for the NaTFA and polymer solutions. Matrix solution was deposited in 0.5 µm aliquots on the sample plate wells and allowed to dry before 0.5 µm of electrolyte/sample solution was deposited on top of the matrix and dried in air.

### Nuclear Magnetic Resonance

1H Nuclear Magnetic Resonance (NMR) experiments were performed on a Bruker Avance‐500 MHz NMR Spectrometer. Dry samples were dissolved in D_2_O at 10 mg mL^−1^. The linear polymer samples prepared in CDCl_3_ were diluted to 10 mg mL^−1^ with additional CDCl_3_.

### APS/TEMED Gel Degradation

For preparing 5 wt.% thiol–maleimide hydrogels for APS/TEMED degradation, LAP was excluded from the formulation and 1 µm polystyrene FluoSpheres (ThermoFisher Scientific) were added at a final concentration of 0.002 wt.% solids. Hydrogels were equilibrated in 0.2 m TEMED (Bio‐Rad) after 20 min of crosslinking. For degradation, these gels were supplemented with an equal volume of PBS containing either no APS or 0.2 m APS (Bio‐Rad) for radical generation (final concentration 0.1 m APS/TEMED for +APS and 0 m APS/0.1 m TEMED for –APS). Time‐lapse imaging was performed on a Nikon Eclipse Ti2 microscope with a CFI Plan Apochromat Lambda D 4X objective (NA = 0.2).

### Fluorescence Recovery After Photobleaching (FRAP)

Thiol–maleimide hydrogels of 5 wt.% were prepared as described above with 1 wt.% LAP. Gels were cast as 8 µL droplets in a 96‐well plate. After 20 min of crosslinking, the hydrogels were equilibrated with 2 µm calcein solution containing 1 wt.% LAP. For control experiments, LAP was not included in the calcein solution. FRAP recovery measurements, photobleaching, and photodegradation were performed on a Nikon A1R laser scanning confocal microscope equipped with a CFI Plan Apochromat Lambda D 4X objective (NA = 0.2). FRAP measurements were acquired in a circular region of interest (ROI) of 50 µm radius with a 15 s baseline, 5 s bleach (488 nm laser, 100% power), and 90 s recovery. For irradiated samples (10 s exposure, 405 nm laser light at 100% power), a square ROI with 400 µm edges was patterned and allowed 10 min for fluorophore equilibration before the FRAP ROI was positioned in the center of the 405 nm‐patterned region.

### Laser Micropatterning

Photopatterning experiments were performed on crosslinked hydrogels synthesized between a glass slide and cover slip with 250 µm spacers. A Zeiss LSM 710 NLO confocal microscope was used to pattern shapes in the hydrogels using 405 nm light (100% power, 0.42 µm pixel size, 0.5 µs pixel dwell, 16 scans with line averaging). Custom.ovl files were prepared using a MATLAB script, and custom binary.tif masks were prepared in Affinity Designer.

## Conflict of Interest

The authors declare no conflict of interest.

## Supporting information

Supporting Information

## Data Availability

The data that support the findings of this study are available from the corresponding author upon reasonable request.

## References

[1] M. J. Majcher , T. Hoare , in Functional Biopolymers, (Eds.: M. A. Jafar Mazumder , H. Sheardown , A. Al‐Ahmed ), Springer International Publishing, Berlin, Germany, 2019, pp. 453–490.

[2] Z. Xu , K. M. Bratlie , ACS Biomater. Sci. Eng. 2018, 4, 2276. 33435096 10.1021/acsbiomaterials.8b00230

[3] D. P. Nair , M. Podgórski , S. Chatani , T. Gong , W. Xi , C. R. Fenoli , C. N. Bowman , Chem. Mater. 2013, 26, 724.

[4] Y. Fu , W. J. Kao , J. Biomed. Mater. Res., Part A 2011, 98A, 201. 10.1002/jbm.a.33106 PMC4529490 21548071

[5] L. E. Jansen , L. J. Negrón‐Piñeiro , S. Galarza , S. R. Peyton , Acta Biomater. 2018, 70, 120. 29452274 10.1016/j.actbio.2018.01.043 PMC5871581

[6] N. J. Darling , Y.‐S. Hung , S. Sharma , T. Segura , Biomaterials 2016, 101, 199. 27289380 10.1016/j.biomaterials.2016.05.053

[7] S. D. Fontaine , R. Reid , L. Robinson , G. W. Ashley , D. V. Santi , Bioconjugate Chem. 2015, 26, 145. 10.1021/bc5005262 25494821

[8] J. Lou , D. J. Mooney , Nat Rev Chem 2022, 1. 37117490 10.1038/s41570-022-00420-7

[9] M. P. Lutolf , J. L. Lauer‐Fields , H. G. Schmoekel , A. T. Metters , F. E. Weber , G. B. Fields , J. A. Hubbell , Proc. Natl. Acad. Sci. 2003, 100, 5413. 12686696 10.1073/pnas.0737381100 PMC154359

[10] J. L. West , J. A. Hubbell , Macromolecules 1999, 32, 241.

[11] A. S. Gobin , J. L. West , FASEB J. 2002, 16, 751. 11923220 10.1096/fj.01-0759fje

[12] S. C. Owen , M. S. Shoichet , J. Biomed. Mater. Res., Part A 2010, 94A, 1321. 10.1002/jbm.a.32834 20597126

[13] A. Gennari , J. Wedgwood , E. Lallana , N. Francini , N. Tirelli , Tetrahedron 2020, 76, 131637.

[14] M. P. Lutolf , J. A. Hubbell , Nat. Biotechnol. 2005, 23, 47. 15637621 10.1038/nbt1055

[15] J. H. Galarraga , A. P. Dhand , B. P. Enzmann III , J. A. Burdick , Biomacromolecules 2023, 24, 413. 36516973 10.1021/acs.biomac.2c01218 PMC10928645

[16] S. M. Kroger , L. Hill , E. Jain , A. Stock , P. J. Bracher , F. He , S. P. Zustiak , Macromol. Biosci. 2020, 20, 2000085. 10.1002/mabi.202000085 32734673

[17] A. Southan , M. Mateescu , V. Hagel , M. Bach , C. Schuh , C. Kleinhans , P. J. Kluger , S. Tussetschläger , I. Nuss , T. Haraszti , S. V. Wegner , J. P. Spatz , H. Boehm , S. Laschat , G. E. M. Tovar , Macromol. Chem. Phys. 2013, 214, 1865.

[18] F.‐Y. Lin , C.‐C. Lin , ACS Macro Lett. 2021, 10, 341. 35549061 10.1021/acsmacrolett.1c00056 PMC10319469

[19] Y. Hao , C.‐C. Lin , J. Biomed. Mater. Res., Part A 2014, 102, 3813. 10.1002/jbm.a.35044 24288169

[20] J. L. Pelloth , P. A. Tran , A. Walther , A. S. Goldmann , H. Frisch , V. X. Truong , C. Barner‐Kowollik , Adv. Mater. 2021, 33, 2102184. 10.1002/adma.202102184 34365684

[21] T. E. Brown , K. S. Anseth , Chem. Soc. Rev. 2017, 46, 6532. 28820527 10.1039/c7cs00445a PMC5662487

[22] V. X. Truong , K. M. Tsang , G. P. Simon , R. L. Boyd , R. A. Evans , H. Thissen , J. S. Forsythe , Biomacromolecules 2015, 16, 2246. 26056855 10.1021/acs.biomac.5b00706

[23] F. Amir , K. P. Liles , A. O. Delawder , N. D. Colley , M. S. Palmquist , H. R. Linder , S. A. Sell , J. C. Barnes , ACS Appl. Mater. Interfaces 2019, 11, 24627. 31251567 10.1021/acsami.9b08853

[24] A. M. Kloxin , A. M. Kasko , C. N. Salinas , K. S. Anseth , Science 2009, 324, 59. 19342581 10.1126/science.1169494 PMC2756032

[25] F. M. Yavitt , B. E. Kirkpatrick , M. R. Blatchley , K. F. Speckl , E. Mohagheghian , R. Moldovan , N. Wang , P. J. Dempsey , K. S. Anseth , Sci. Adv. 9, eadd5668. 36662859 10.1126/sciadv.add5668 PMC9858500

[26] R. Y. Tam , L. J. Smith , M. S. Shoichet , Acc. Chem. Res. 2017, 50, 703. 28345876 10.1021/acs.accounts.6b00543

[27] W. Huang , X. Wu , X. Gao , Y. Yu , H. Lei , Z. Zhu , Y. Shi , Y. Chen , M. Qin , W. Wang , Y. Cao , Nat. Chem. 2019, 11, 310. 30718898 10.1038/s41557-018-0209-2

[28] F. Dénès , C. H. Schiesser , P. Renaud , Chem. Soc. Rev. 2013, 42, 7900. 23828205 10.1039/c3cs60143a

[29] M. Huo , J. G. Hu , D. R. Clarke , Macromolecules 2023, 56, 9107.

[30] K. Cheng , A. Chortos , J. A. Lewis , D. R. Clarke , ACS Appl. Mater. Interfaces 2022, 14, 4552. 35006669 10.1021/acsami.1c22287

[31] H. Zipse , in Radicals in Synthesis I, (Ed.: A. Gansäuer ), Springer, Berlin Heidelberg, Berlin, Heidelberg 2006, pp. 163–189.

[32] A. R. Killaars , J. C. Grim , C. J. Walker , E. A. Hushka , T. E. Brown , K. S. Anseth , Adv. Sci. (Weinh) 2019, 6, 1801483. 30775233 10.1002/advs.201801483 PMC6364489

[33] Y. Gao , K. Peng , S. Mitragotri , Adv. Mater. 2021, 33, 2006362. 10.1002/adma.202006362 33988273

[34] B. D. Fairbanks , M. P. Schwartz , C. N. Bowman , K. S. Anseth , Biomaterials 2009, 30, 6702. 19783300 10.1016/j.biomaterials.2009.08.055 PMC2896013

[35] G. J. Berg , T. Gong , C. R. Fenoli , C. N. Bowman , Macromolecules 2014, 47, 3473.

[36] SDBSWeb: , https://sdbs.db.aist.go.jp, National Institute of Advanced Industrial Science and Technology. (accessed: January 2024).

[37] D. Smith , J. Myers , C. Kaplan , C. Goodman‐Strauss , arXiv, 2023, arXiv.2303.10798.

