# Supplementary material for: Radical‐Mediated Degradation of Thiol–Maleimide Hydrogels
Source: Adv Sci (Weinh). 2024 Apr 6;11(25):2402191. doi: 10.1002/advs.202402191 (PMC11220706; doi:10.1002/advs.202402191)
Supplement: Supplementary file 1 — Supporting Information [file ADVS-11-2402191-s001.pdf]

ADVANCED SCIENCE
Open Access

Supporting Information

for *Adv. Sci.*, DOI 10.1002/advs.202402191

Radical-Mediated Degradation of Thiol–Maleimide Hydrogels

*Tayler S. Hebner, Bruce E. Kirkpatrick, Benjamin D. Fairbanks, Christopher N. Bowman, Kristi S. Anseth and Danielle S.W. Benoit**

Supporting Information

Radical-Mediated Degradation of Thiol-Maleimide Hydrogels

*Tayler S. Hebner, Bruce E. Kirkpatrick, Benjamin D. Fairbanks, Christopher N. Bowman, Kristi S. Anseth, Danielle S.W. Benoit**

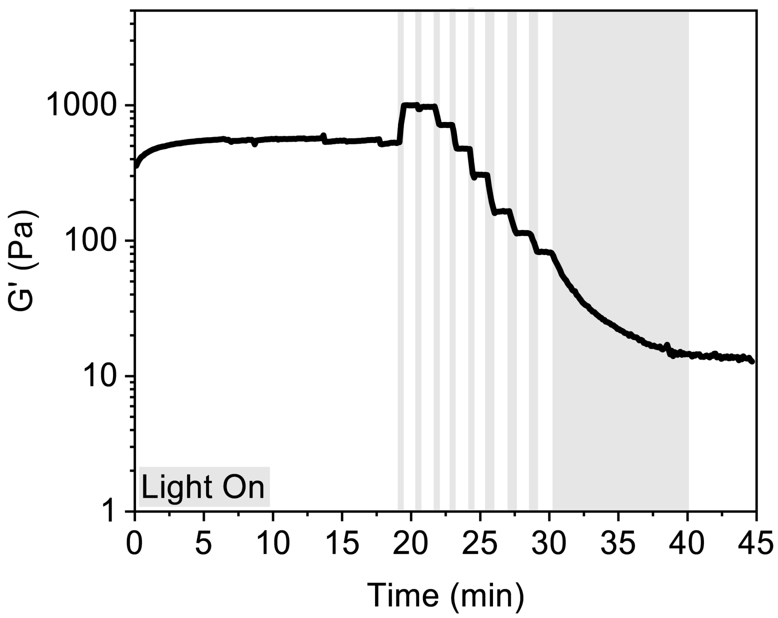

Figure S1. In-situ oscillatory shear rheology (1% strain, 1 rad/s) monitoring storage modulus during Michael addition in the dark for 20 minutes, followed by five intervals of alternating 15 seconds of UV light exposure (365 nm, 5 mW/cm^2) and 1 minute in the dark, then three intervals of alternating 30 seconds of UV exposure and 1 minute in the dark, then one final UV exposure 9 minutes followed by 5 minutes in the dark. Hydrogels used in this experiment were synthesized using 4-arm 20 kDa PEG-maleimide with 2 kDa PEG-dithiol at 1:1 molar ratio of thiol:maleimide. Macromers were mixed at 5 wt% solids in PBS with 0.5 wt% LAP.

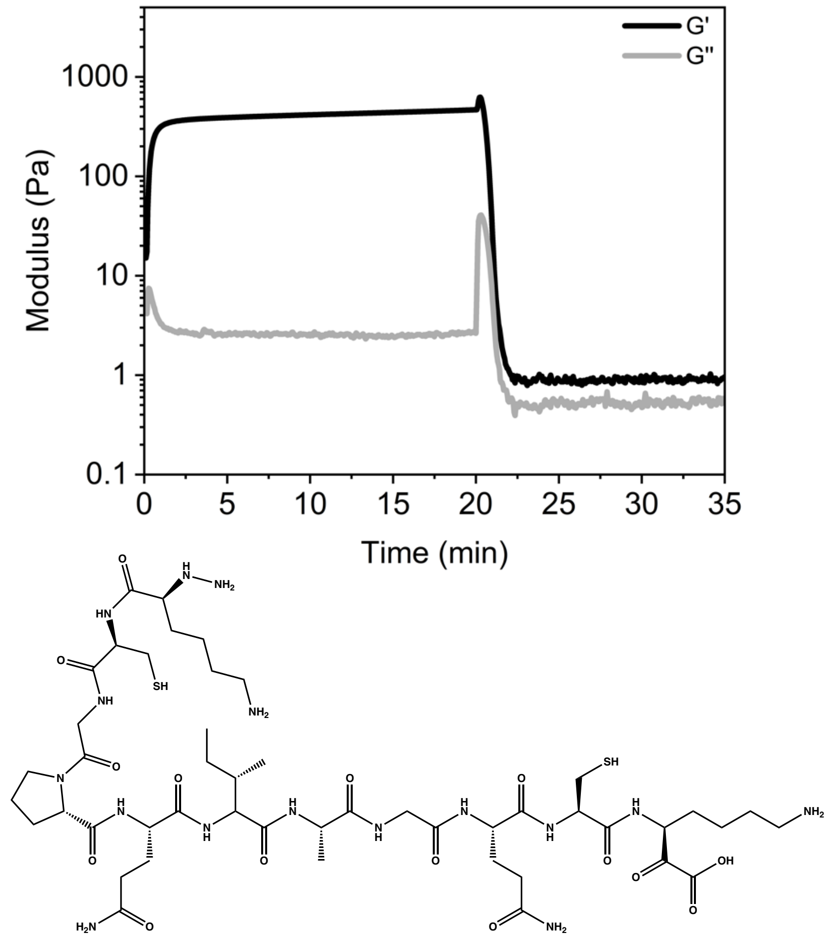

Figure S2. In-situ oscillatory shear rheology (1% strain, 1 rad/s) monitoring storage modulus (black) and loss modulus (gray) during Michael addition in the dark for 20 minutes, followed by 10 minutes of UV light exposure (365 nm, 5 mW/cm^2) and 5 minutes in the dark. Hydrogels used in this experiment were synthesized using 4-arm 20 kDa PEG-maleimide with a dicysteine functionalized peptide (KCGPQGIAGQCK) at 1:1 molar ratio of thiol:maleimide. Macromers were mixed at 5 wt% solids in PBS with 0.5 wt% LAP. The structure of the peptide used in these experiments is shown below the rheology data.

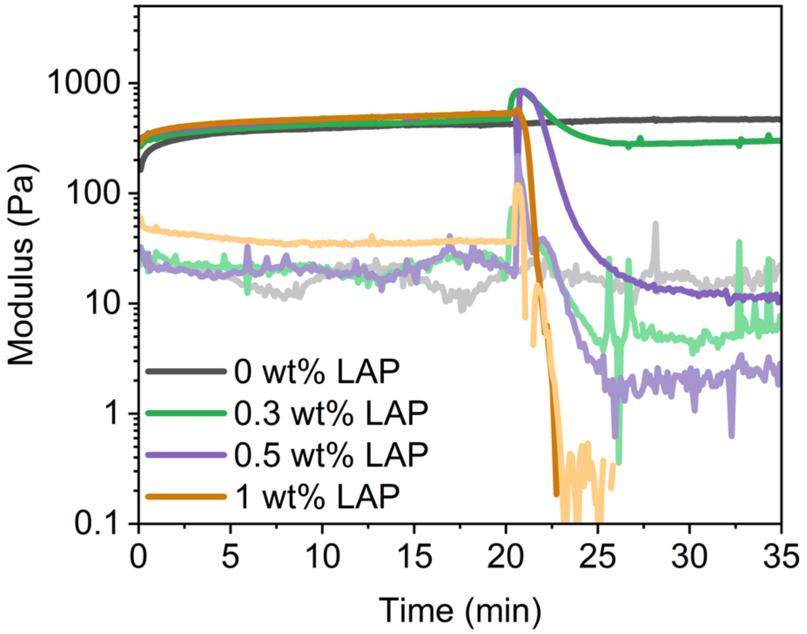

Figure S3. In-situ oscillatory shear rheology (1% strain, 1 rad/s) monitoring storage modulus (dark traces) and loss modulus (light traces) during Michael addition in the dark for 20 minutes, followed by 10 minutes of UV light exposure (365 nm, 5 mW/cm^2) and 5 minutes in the dark. Hydrogels used in this experiment were synthesized using 4-arm 20 kDa PEG-maleimide with 2 kDa PEG-dithiol at 1:1 molar ratio of thiol:maleimide. Macromers were mixed at 5 wt% solids in PBS with LAP concentrations of 0 (gray), 0.3 (green), 0.5 (purple), or 1 (orange) wt%.

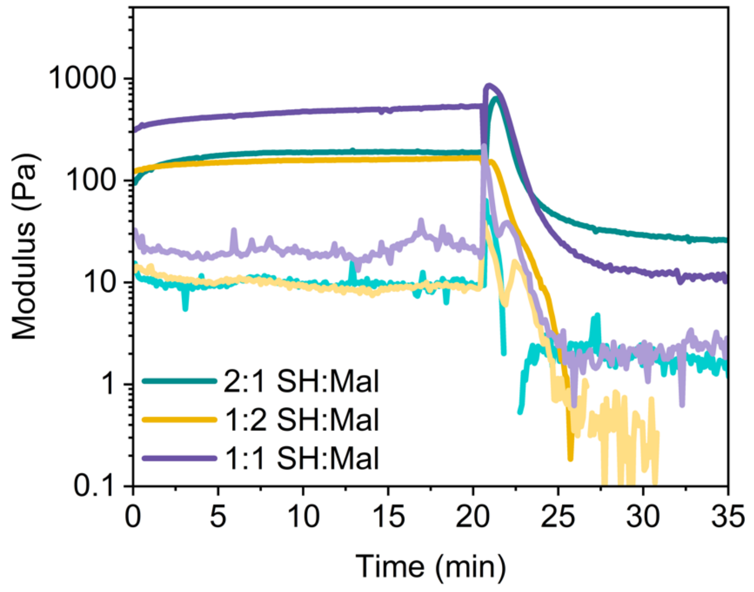

Figure S4. In-situ oscillatory shear rheology (1% strain, 1 rad/s) monitoring storage modulus (dark traces) and loss modulus (light traces) during Michael addition in the dark for 20 minutes, followed by 10 minutes of UV light exposure (365 nm, 5 mW/cm^2) and 5 minutes in the dark. Hydrogels used in this experiment were synthesized using 4-arm 20 kDa PEG-maleimide with 2 kDa PEG-dithiol at 2:1 (green), 1:1 (purple), or 1:2 (yellow) molar ratio of thiol:maleimide. Macromers were mixed at 5 wt% solids in PBS with a LAP concentration of 0.5 wt%.

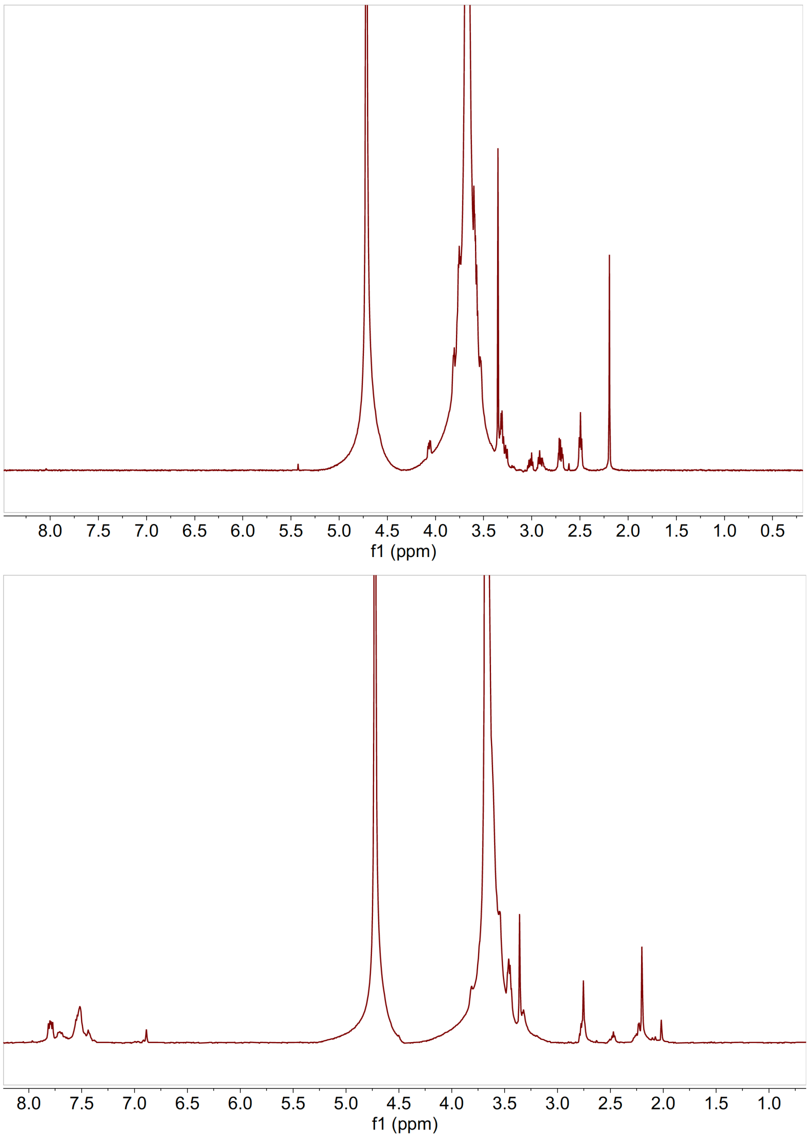

Figure S5. ^{1}H NMR spectra for linear polymers after Michael addition (top) and radical-mediated degradation (bottom). Polymers were synthesized using 1 kDa monofunctional PEG-maleimide and 2 kDa PEG-dithiol at 1:1 molar ratio of thiol:maleimide in PBS with 9 mM concentration of functional groups. The polymers subjected to Michael addition only were synthesized without LAP while the degraded polymers were synthesized in the presence of 0.5 wt% LAP. Michael addition was carried out in solution for 20 minutes for both samples, and the sample containing LAP was then exposed to UV light (365 nm, 5 mW/cm^2) for 15 minutes. All samples were lyophilized and dissolved at 10 mg/mL in D_2O for NMR analysis.

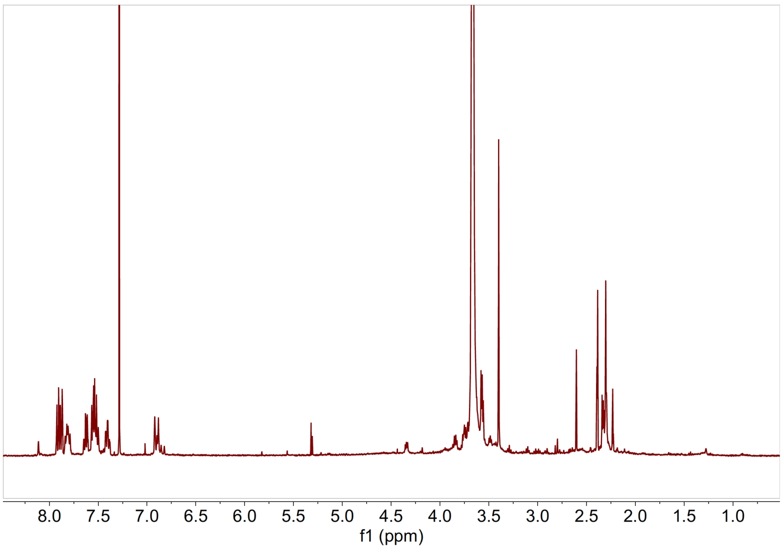

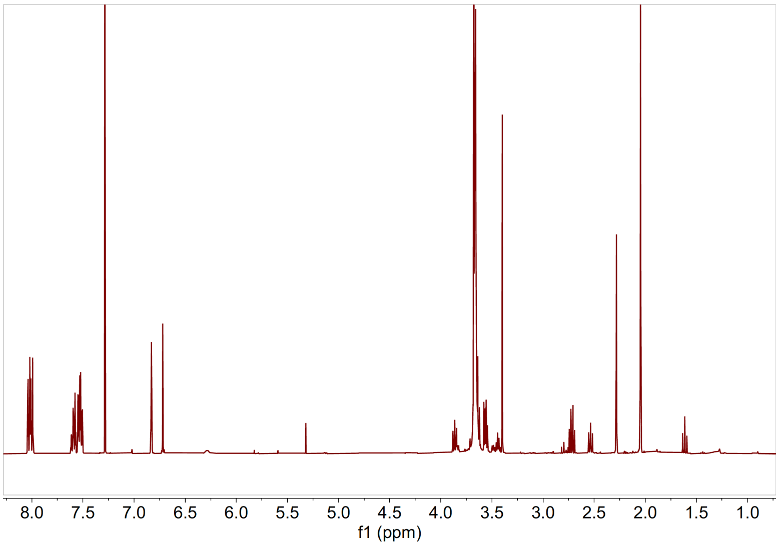

Figure S6. ^{1}H NMR spectra for linear polymers after Michael addition only (top) and after radical-mediated degradation (bottom). Polymers were synthesized using 1 kDa monofunctional PEG-maleimide and 2 kDa PEG-dithiol at 1:1 molar ratio of thiol:maleimide in CDCl3 with 9 mM concentration of functional groups. The polymers were synthesized in the presence of 0.5 wt% TPO. Michael addition was carried out in solution for 20 minutes for both samples, and the sample containing LAP was then exposed to UV light (365 nm, 5 mW/cm^2) for 15 minutes. All samples were lyophilized and dissolved at 10 mg/mL in D2O for NMR analysis. Peak changes in the region of ~6.5 ppm to 8.0 ppm are attributed to soluble initiator fragments. Peak changes in the region of ~4.2 ppm to 4.5 ppm are attributed to disappearance of the Michael adduct.

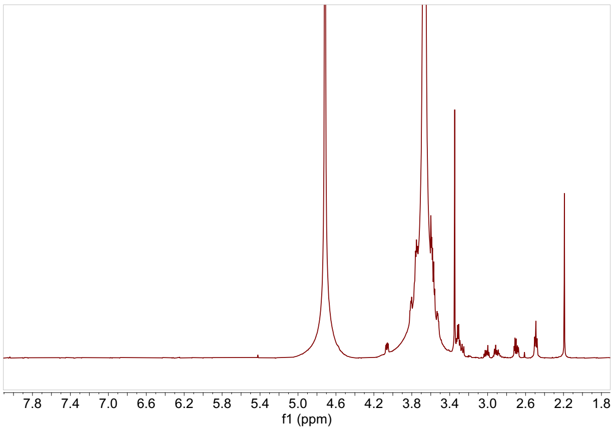

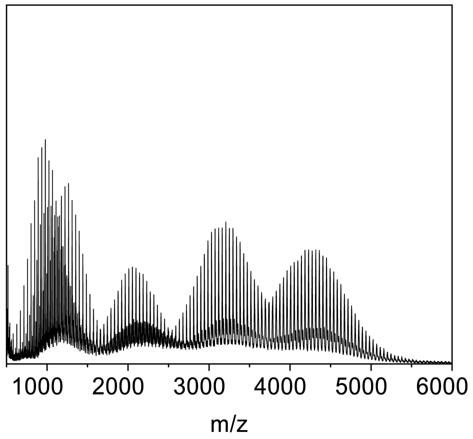

Figure S7. ^{1}H NMR (top) and MALDI (bottom) spectra of linear polymers that were exposed to UV light (365 nm, 5 mW/cm^2, 15 min) with no photoinitiator in solution. Polymers were synthesized via Michael addition for 20 minutes using 1 kDa monofunctional PEG-maleimide and 2 kDa PEG-dithiol at 1:1 molar ratio of thiol:maleimide in PBS with 9 mM concentration of functional groups.

Estimation of light attenuation, radical concentration over time, and reverse gel point

Minimal light attenuation was confirmed in our samples (i.e., that incident photons reached the bottom of the sample during irradiation) using known values of sample depth, wavelength, intensity, a literature value of LAP absorptivity,[42] and a conservative estimate of quantum yield.

Beer's law was used to estimate attenuation at a depth of *t* = 300 µm with 0.5 and 1 wt% LAP. Attenuation is modeled as an exponential decay that varies with absorbance, photoinitiator concentration, and sample thickness: $I = I_0 \mathrm{e}^{-(\epsilon * [\mathrm{PI}] * t)}$ or $\mathrm{attenuation} = 1 - \mathrm{e}^{-(\epsilon * [\mathrm{PI}] * d)}$.

The absorptivity of LAP was estimated to be 225 $cm^{-1}M^{-1}$ from ref. [41]. With known [PI] of 0.5 or 1 wt% (17 or 34 mM) and a thickness (*d*) of 300 µm (no thicker than any of our samples for photoinitiated degradation), attenuation is less than ~20% through the thickness of any of our samples (0.205 for 1 wt% LAP, 0.108 for 0.5 wt% LAP). As such, we do not consider attenuation in our other calculations.

To calculate the concentration of radicals generated at varied time, the following calculations were performed:

Absorbed photon flux: $\phi = \frac{I * \epsilon * \lambda}{h * c * N}$ where *I* is intensity (5 mW/cm^2), $\lambda = 365$ nm, and *h*, *c*, and *N* respectively represent Planck's constant, the speed of light, and Avogadro's number.

PI (unreacted initiator) concentration over time: $[\mathrm{PI}] = [\mathrm{PI}]_0 * e^{(-\phi * \Phi * t)}$ where Φ is a quantum yield of 0.5 and *t* is time, which was calculated from 0 to 3600 seconds in 20 second intervals. With these estimates, all LAP is consumed after 3600 seconds (1 hour) of 365 nm irradiation at 5 mW/cm^2.

Radical production rate: $\mathrm{I}_\mathrm{r}^* = [\mathrm{PI}] * \phi * \Phi$ (I_r^* is in M/s).

Cumulative radicals generated: $\mathrm{I}_\mathrm{i}^* = \sum_{t=0}^{i} \mathrm{I}_\mathrm{r}^* * t$.

Combining the equations for absorbed photon flux, initiator concentration over time, radical production rate, and cumulative radicals generated, we estimate that **5.02 mmol** of radicals are generated within 180 seconds of irradiation with the given conditions and 0.5 wt% LAP. It is important to note that radical recombination or other potential sources of non-productive radicals were not accounted for in these estimates.

Finally, to estimate the reverse gel point: the hydrogel formulation used throughout our studies includes 4.5 wt% 4-arm 20 kDa PEG-maleimide (A) and stoichiometric dithiol (B) ($r = 1$, $f_A = 4$, $f_B = 2$), meaning that 9 mmol crosslinks (TM) are formed with complete conversion. Using Flory-Stockmayer theory of percolation, the concentration of crosslinks that must be cleaved to reach the reverse gel point can be estimated as:

$$\mathrm{TM} * \left(1 - \sqrt{\frac{1}{r * (f_A - 1) * (f_B - 1)}}\right) = 9\ \mathrm{mmol} * \left(1 - \sqrt{\frac{1}{3}}\right) = \mathbf{3.8\ mmol}$$

Considering that only a fraction of the estimated radicals generated within a few minutes of irradiation (of 0.5 wt% LAP with 365 nm light at 5 mW/cm^2) would be necessary to reach the calculated reverse gel point in our system, we infer that the degradation reaction is not 100% efficient, particularly in the presence of other potentially radical-scavenging species (e.g., unreacted maleimide).
